# Determinants of magnesium sulphate use in women hospitalized at <29 weeks with severe or non-severe pre-eclampsia

**DOI:** 10.1371/journal.pone.0189966

**Published:** 2017-12-22

**Authors:** Dane A. De Silva, Lily Proctor, Peter von Dadelszen, Meghan McCoach, Tang Lee, the Canadian Perinatal Network (CPN) Collaborative Group, Laura A. Magee

**Affiliations:** 1 Department of Obstetrics & Gynaecology, University of British Columbia, Vancouver, Canada; 2 Department of Women and Children’s Health, St Thomas’ Hospital, London, United Kingdom; 3 School of Life Course Sciences, Faculty of Life Sciences and Medicine, King’s College London, London, United Kingdom; Stellenbosch University, SOUTH AFRICA

## Abstract

**Objective:**

Magnesium sulphate is recommended by international guidelines to prevent eclampsia among women with pre-eclampsia, especially when it is severe, but fewer than 70% of such women receive magnesium sulphate. We aimed to identify variables that prompt Canadian physicians to administer magnesium sulphate to women with pre-eclampsia.

**Methods:**

Data were used from the Canadian Perinatal Network (2005–11) of women hospitalized at <29 weeks’ who were thought to be at high risk of delivery due to pre-eclampsia (using broad Canadian definition). Unadjusted analyses of relative risks were estimated directly and population attributable risk percent (PAR%) calculated to identify variables associated with magnesium sulphate use. A multivariable model was created and a generalized estimating equation was used to estimate the adjusted RR that explained magnesium sulphate use in pre-eclampsia. The adjusted PAR% was estimated by bootstrapping.

**Results:**

Of 631 women with pre-eclampsia, 174 (30.1%) had severe pre-eclampsia, of whom 131 (75.3%) received magnesium sulphate. 457 (69.9%) women had non-severe pre-eclamspia, of whom 291 (63.7%) received magnesium sulphate. Use of magnesium sulphate among women with pre-eclampsia could be attributed to the following clinical factors (PAR%): delivery for ‘adverse conditions’ (48.7%), severe hypertension (21.9%), receipt of antenatal corticosteroids (20.0%), maternal transport prior to delivery (9.9%), heavy proteinuria (7.8%), and interventionist care (3.4%).

**Conclusions:**

Clinicians are more likely to administer magnesium sulphate for eclampsia prophylaxis in the presence of more severe maternal clinical features, in addition to concomitant antenatal corticosteroid administration, and shorter admission to delivery periods related to transport from another institution or plans for interventionist care.

## Introduction

Magnesium sulphate is effective for treatment of eclampsia [1]. The Magpie Trial demonstrated that magnesium sulphate could halve the rate of seizures among women with pre-eclampsia [2]. Magnesium sulphate was equally effective for severe and non-severe pre-eclampsia, although treatment of women with non-severe pre-eclampsia required a high number-needed-to-treat (NNT) to prevent one seizure (i.e., 100 vs. 50) and at a higher cost (US $21,202 vs. $12,942) compared to severe pre-eclampsia [3]. In well-resourced settings, this has led to recommendations to administer magnesium sulphate to all women with ‘severe’ pre-eclampsia, and consider doing so to women with non-severe disease. In under-resourced settings, similar recommendations have been made by the World Health Organization (WHO), citing that magnesium sulphate may not be available for all women [4].

In the seminal Magpie Trial, the definition of severe pre-eclampsia was based on severe hypertension and heavier proteinuria (≥3+), or less severe hypertension associated with findings of ‘imminent eclampsia’ for which there is no standard definition but is usually interpreted as central nervous system symptoms or hyperreflexia [2]. This definition of severe disease does not align well with current international definitions between which there is also substantial variability from country to country, and within countries over time [5].

In Canada, the 1997 national pregnancy hypertension guidance, pre-eclampsia was broadly defined as gestational hypertension with proteinuria or an adverse condition(s) that consisted of relevant maternal symptoms, signs, or abnormal laboratory tests, or relevant fetal manifestations; ‘severe’ pre-eclampsia was not defined [6–8]. In the 2008 update, ‘severe’ pre-eclampsia was defined as gestational hypertension with proteinuria *and* an ‘adverse condition(s)’ as defined previously [9]. In the 2014 update, ‘severe’ pre-eclampsia was defined according to the presence of an indication for delivery—a severe complication(s) for the mother or fetus [10,11]. Magnesium sulphate was recommended for all women in 1997, and then in 2008 and 2014, all women with ‘severe’ pre-eclampsia, with consideration to be given to also administering magnesium sulphate to women with ‘non-severe’ disease.

In the international Pre-eclampsia Integrated Estimate of RiSk (PIERS) study that aimed to find predictors of adverse maternal outcome among women admitted to hospital with pre-eclampsia, magnesium sulphate was administered to only 62% of 261 women who were both hospitalized with pre-eclampsia and suffered an adverse maternal outcome that would meet any international definition of ‘severe’ disease, suggesting that clinicians may be using criteria other than strict definitions of ‘severe’ or ‘non-severe’ disease to guide therapy [12]. The aim of our study was to identify factors that influence magnesium sulphate administration to women with pre-eclampsia in Canadian tertiary perinatal centres.

## Materials and methods

The Canadian Perinatal Network (CPN) is a database of women admitted to 16 tertiary care centres in Canada (August 2005 to March 2011) at 22^+0^ to 28^+6^ weeks gestation because of threatened preterm birth. These women were admitted with one or more of: spontaneous preterm labour with contractions, preterm pre-labour rupture of membranes (PPROM), short cervix without contractions, prolapsing membranes, gestational hypertension, intrauterine fetal growth restriction (IUGR), or antepartum haemorrhage. This study was approved centrally as a quality assurance project at the University of British Columbia (H05-70359) and at each participating site’s Research Ethics Board. Details of the CPN study have been published previously [13].

In this analysis, we included women who presented to a participating CPN site with pre-eclampsia/eclampsia (before 29 weeks) as their primary indication for admission. Pre-eclampsia was defined broadly as gestational hypertension with proteinuria, or one or more of relevant pre-eclampsia maternal symptoms, signs or abnormal laboratory tests; this definition was consistent with the 1997, 2008, and 2014 national guidance in Canada, as published by the Society of Obstetricians and Gynaecologists of Canada (SOGC) [5–11].

The primary outcome was magnesium sulphate administration for pre-eclampsia/eclampsia, antenatally or postnatally (**S1 File**).

Descriptive statistics were used to compare the characteristics of women who received magnesium sulphate, with those who did not, using the Chi-square (categorical variables) and Mann-Whitney U tests (continuous variables), with a p-value <0.05 considered to be statistically significant. As the primary outcome of magnesium sulphate use was a common occurrence, univariable analyses of relative risks (RR) were estimated directly using generalized linear models with a binomial distribution and a log link instead of calculating odds ratios (OR) (as an OR would be expected to overestimate the RR with a common outcome).

For inclusion in the multivariable model, we tested candidate variables that were either associated with magnesium sulphate use at p<0.10 or differed among women treated with magnesium sulphate (vs. those who were not) by an absolute amount that could be clinically important and identify lost therapeutic opportunities that could be addressed to improve magnesium sulphate use and outcomes. As such, variables that occurred infrequently (among <5 women) were not included. Continuous variables, such as maternal age and blood pressure, were collapsed into meaningful categories, and some inter-related outcomes were combined (e.g., fetal syndrome of pre-eclampsia) in order to avoid problems of model convergence. The variables were reviewed to identify those with the highest RR and eliminate those that were likely to be highly intercorrelated with other included variables to create the most parsimonious model. The final list of variables was determined through expert opinion from obstetric medicine, obstetrics, and epidemiology.

A final generalized estimating equation was used to account for the multicentre design of CPN and estimate the adjusted RR that explained magnesium sulphate use. Adjusted Population Attributable Risk percent (PAR%) was calculated for each variable in the model to identify determinants of magnesium sulphate use. PAR% for a variable was interpreted as the proportion of magnesium sulphate use that was attributable to that variable, noting that the PAR% for different variables are not additive [14]. The 95% CI for the adjusted PAR% were estimated by bootstrapping methods [15].

All statistical analyses were performed using R statistical software (www.r-project.org).

In sensitivity analyses, we explored the impact of: (i) adding as a determinant pre-eclampsia severity defined according to the 2014 SOGC HDP guidelines [9,10], defined in detail in **Table A in S2 File**; and (ii) excluding variables that were assumed to have been present prior to magnesium sulphate use, but that were not time-stamped: (a) severe hypertension that was replaced by severe hypertension therapy (with parenteral hydralazine or labetalol, or nifedipine capsules or intermediate-acting tablets) that was time-stamped, and (b) severe hypertension and heavy proteinuria; and (iii) restricting the analysis to intrapartum/postpartum therapy to examine the impact of variables that were antepartum but not time-stamped.

## Results

There were 631 eligible women who were at 22^+0^ and 28^+6^ weeks gestation when admitted to one of 16 CPN participating tertiary perinatal centres for pre-eclampsia care. 422 (66.9%) women received magnesium sulphate for eclampsia prophylaxis.

**Table 1** presents the baseline maternal and pregnancy characteristics of the women included. Women who received magnesium sulphate (compared with those who did not) differed according to most admission maternal and pregnancy characteristics. These women were more frequently: younger in age, nulliparous, had a history of gestational hypertension, and demonstrated more severe maternal clinical features of pre-eclampsia, in terms of higher BP, heavier proteinuria, and more frequent serious maternal end-organ complications (for details, see **Table B in S2 File**). Of note, these women treated with magnesium sulphate were less likely to have fetal manifestations and stillbirth. They also had shorter admission to delivery intervals and delivered at an earlier gestational age and more often by Caesarean.

**Table 1:** Demographic and clinical characteristics of women with severe/non-severe pre-eclampsia according to MgSO4 use (N = 631).

	MgSO4 use N = 422	No MgSO4 use N = 209	Unadjusted RR (95% CI)	p-value
**Demographic and clinical characteristics in index pregnancy**				
Maternal age (yr)	30.0 [26.0, 35.0]	33.0 [29.0, 37.0]	-	<0.001
≤24	76 (18.0%)	17 (8.1%)	1.26 (1.09, 1.45)	
25–29	108 (25.6%)	44 (21.1%)	1.09 (0.94, 1.27)	
30–34	115 (27.3%)	62 (29.7%)	Reference	
≥35	123 (29.1%)	86 (41.1%)	0.91 (0.77, 1.06)	
Pre-existing medical conditions				
Pre-existing hypertension	87 (20.6%)	57 (27.3%)	0.88 (0.76, 1.02)	0.076
Diabetes mellitus	9 (2.1%)	10 (4.8%)	0.70 (0.44, 1.13)	0.113
Venous thromboembolism	2 (0.5%)	4 (1.9%)	-	0.097
Nulliparous	298 (70.6%)	121 (57.9%)	1.22 (1.07, 1.38)	0.002
Singleton pregnancy	396 (93.8%)	182 (87.1%)	1.40 (1.06, 1.85)	0.006
Previous gestational hypertension	60 (14.2%)	52 (24.9%)	0.84 (0.69, 1.02)	0.001
Gestational age at enrolment	26.9 [25.3, 28.0]	26.6 [25.1, 27.9]	-	0.098
Blood pressure				
Peak sBP	180 [168, 193]	168 [158, 180]	-	<0.001
Peak dBP	107 [100, 114]	100 [95, 109]	-	<0.001
sBP ≥160 or dBP ≥110 mmHg	388 (91.9%)	157 (75.1%)	1.80 (1.38, 2.35)	<0.001
Proteinuria	408 (96.7%)	190 (90.9%)	1.61 (1.08, 2.40)	0.004
≥3+ on dipstick or ≥3g/d	335 (79.4%)	127 (60.8%)	1.41 (1.20, 1.65)	<0.001
**Maternal Interventions prescribed**				
Bedrest	311 (73.7%)	165 (78.9%)	0.91 (0.81, 1.03)	0.179
Interventionist care^a^	111 (26.3%)	15 (7.2%)	1.43 (1.30, 1.57)	<0.001
Maternal transport prior to delivery	268 (63.5%)	80 (38.3%)	1.42 (1.25, 1.60)	<0.001
Any antihypertensive therapy	388 (91.9%)	187 (89.5%)	1.11 (0.89, 1.38)	0.380
Antenatal corticosteroids	376 (89.1%)	156 (74.6%)	1.52 (1.22, 1.89)	<0.001
**Progress after admission & outcomes**				
Severe maternal complications (one/more)^b^	163 (38.6%)	59 (28.2%)	1.16 (1.04, 1.29)	0.013
Fetal syndrome of pre-eclampsia (one/more)	271 (64.2%)	149 (71.3%)	0.90 (0.81, 1.01)	0.09
Abnormal umbilical artery Doppler	116 (27.5%)	72 (34.4%)	-	0.089
Oligohydramnios	51 (12.1%)	29 (13.9%)	-	0.611
Birthweight <10^th^ centile	130 (30.8%)	92 (44.0%)	-	0.001
Stillbirth	48 (11.4%)	28 (13.4%)	-	0.545
Delivered on 1^st^ admission	372 (88.2%)	143 (68.4%)	-	<0.001
Indication for delivery				
Uncontrolled hypertension	169 (40.0%)	81 (38.8%)	1.02 (0.91, 1.14)	0.821
Maternal pre-eclampsia symptoms	338 (80.1%)	75 (35.9%)		<0.001
Other maternal signs or abnormal pre-eclampsia lab results	275 (65.2%)	55 (26.3%)	3.33 (2.48, 4.46)	<0.001
Latency, enrolment to delivery (d)	3.0 [1.0, 7.0]	3.0 [11.0, 34.0]	-	<0.001
Gestational age at delivery (wk)	27.7 [26.0, 28.7]	28.7 [27.0, 31.6]	-	<0.001
Mode of delivery				
Vaginal	76 (18.0%)	55 (26.3%)	0.84 (0.72, 0.98)	0.021
Caesarean	346 (82.0%)	155 (74.2%)	1.18 (1.01, 1.38)	0.029
Spontaneous labour	8 (1.9%)	24 (11.5%)	0.49 (0.29, 0.83)	<0.001
Neonatal death prior to or during NICU admission	32 (7.6%)	13 (6.2%)	-	0.644

Data presented as N(%) or median [IQR]

dBP (diastolic blood pressure), NICU (neonatal intensive care unit), sBP (systolic blood pressure), wk (weeks)

^a^ Pregnancies that were not expectantly managed.

^b^ See Table B in S2 File for details of severe maternal complications.

**Table 2** outlines the 15 variables considered for the final model of factors associated with magnesium sulphate use in pre-eclampsia. The following variables were excluded: (i) prior venous thromboembolism (as it was very uncommon); (ii) prior gestational hypertension (as it would not apply to nulliparous women); (iii) gestational age on admission or delivery (as the difference was not clinically significant, and the majority of these women delivered very preterm); (iv) peak systolic and diastolic BP (in favour of severe hypertension); (v) any, as opposed to heavy, proteinuria (because almost all women had some proteinuria and there was a larger difference between groups in heavy proteinuria); (vi) whether this was the woman’s first admission and latency from enrolment to delivery, both of which were accounted for by interventionist care; and (vii) mode of delivery (as the key decision point is the timing of delivery as spontaneous or induced). Also, fetal manifestations of pre-eclampsia were combined into one ‘fetal syndrome’ variable, and delivery for maternal symptoms and signs combined to create a parsimonious model.

**Table 2:** Determinants included in the final model for magnesium sulphate use in all pre-eclampsia^a^.

Determinants	Adjusted RR [95% CI]	PAR% [95% CI]
**Demographic and clinical characteristics in index pregnancy**		
Maternal age (yr)		
≤24	1.11 [1.05, 1.17]	1.42 [-0.55, 2.88]
25–29	1.03 [0.96, 1.11]	0.73 [-3.42, 3.15]
30–34	Reference	Reference
≥35	1.01 [0.92, 1.11]	0.35 [-5.05, 3.95]
Pre-existing hypertension	0.93 [0.86, 0.997]	-1.82 [-4.79, 0.85]
Nulliparity	1.08 [1.00, 1.16]	4.88 [-2.32, 11.61]
Singleton pregnancy	1.23 [0.99, 1.53]	17.38 [-3.34, 38.03]
**Pre-eclampsia severity criteria**		
Severe hypertension (sBP ≥160 or dBP ≥110)	1.34 [1.08, 1.67]	21.92 [9.27, 35.99]
Heavy proteinuria (≥3+ or ≥3.0g/d)	1.12 [1.01, 1.24]	7.82 [0.18, 17.12]
Delivery for maternal symptoms or sign(s) of pre-eclampsia	2.73 [1.30, 5.72]	48.67 [41.40, 56.36]
Severe maternal complications	1.02 [0.95, 1.10]	0.86 [-2.16, 3.86]
Fetal syndrome of pre-eclampsia^b^	0.94 [0.89, 0.99]	-4.58 [-11.28, 2.02]
**Maternal interventions prescribed**		
Maternal transport prior to delivery	1.22 [1.12, 1.32]	9.87 [4.63, 15.00]
Interventionist care	1.21 [1.17, 1.25]	3.41 [1.92, 4.95]
Antenatal corticosteroids	1.31 [1.09, 1.58]	20.01 [9.78, 32.39]
Spontaneous labour initiation	0.72 [0.26, 2.02]	-2.00 [-7.83, 0.48]

BP (blood pressure), dBP (diastolic BP), PAR% (population attributable risk), RR (relative risk), sBP (systolic BP)

^a^ Variables highlighted in yellow demonstrated significant, independent associations with magnesium sulphate use.

^b^ Includes one/more of abnormal Doppler of umbilical artery, oligohydramnios, intrauterine fetal growth restriction, birthweight <10^th^ centile, and stillbirth.

The adjusted RR and PAR% and associated 95% confidence intervals show that there were six factors that were independently associated with magnesium sulphate use. The strongest related to maternal symptoms and signs of maternal disease (PAR% of 48.6%) or severe hypertension (PAR% of 21.9%).

In sensitivity analyses, the final model changed little. The SOGC classification of the severity of pre-eclampsia [i.e., 190 (30.1%) women with ‘severe’ pre-eclampsia among whom 139 (73.2%) received magnesium sulphate, and 441 (69.9%) with non-severe pre-eclampsia of whom 283 (64.2%) received magnesium sulphate] was not independently associated with magnesium sulphate use when added to the final model (**Table C in S2 File**). Replacement of severe hypertension with severe hypertension therapy caused heavy proteinuria (which had been of borderline statistical significance before, but significant) to be dropped, and singleton pregnancy to emerge (that had been of borderline statistical significance before, but not significant) (**Table D in S2 File**). When severe hypertension and heavy proteinuria were excluded, singleton pregnancy again emerged as significant (**Table E in S2 File**). Only when analysis was restricted to antepartum predictors and intrapartum/postpartum magnesium sulphate delivery did maternal transport prior to delivery and interventionist care fall out of the model, but delivery for the fetal syndrome of pre-eclampsia emerged as a significant negative predictor (i.e., of NOT receiving magnesium) (**Table F in S2 File**); the direction of effect was the same in the final model and other sensitivity analyses, but they were not statistically significant. Confidence intervals were wide.

## Discussion

### Summary of results

In a large cohort of women with pre-eclampsia who were hospitalized at <29 weeks to Canadian tertiary perinatal centres, magnesium sulphate was used suboptimally for eclampsia prophylaxis. Even among those with serious maternal complications that constitute indications for delivery and meet all international criteria for ‘severe’ pre-eclampsia, magnesium sulphate was administered in 75.3% (131/174) of cases. Magnesium sulphate for eclampsia prophylaxis was more likely to be administered when: (i) antenatal corticosteroids had also been administered; (ii) delivery was indicated based on maternal symptoms or signs, or there was severe hypertension or heavy proteinuria; or (iii) the clinician had been managing the pre-eclampsia for a shorter period of time and was proceeding with interventionist care. The model was essentially unchanged in sensitivity analyses, including those that added into the model the Canadian definition of ‘severe’ pre-eclampsia, and singleton pregnancy replacing heavy proteinuria in analyses where it was excluded, though both of these are unmodifiable clinical factors.

### How the results compare with the existing literature

Underutilization of magnesium sulphate among women with ‘severe’ pre-eclampsia is consistent with other studies, whether they be single-centre studies in Canada [16] or multicentre international studies [12]. Variation in opinion about what constitutes ‘severe’ pre-eclampsia is reflected in between-protocol variation in 22 Canadian tertiary hospitals and within-hospital differences in protocol and practice [17].

The seminal Magpie Trial that demonstrated the effectiveness of magnesium sulphate for eclampsia prevention when clinicians focussed on treating women with symptoms of ‘imminent eclampsia’, severe hypertension, or heavy proteinuria [2]. Our results suggest that clinicians are heeding maternal symptoms and severe hypertension, but there is room for improvement. Also, clinicians appear to be influenced by additional factors, such as maternal signs other than severe hypertension as indications for delivery, medication (concomitant administration of antenatal corticosteroids), transport prior to delivery, and interventionist care. Further, although singleton pregnancy was not always a significant result, the high PAR% indicates that clinicians may also be influenced by it.

### Strengths and limitations

The strength of this study includes a large population dataset with representation from 16 tertiary centres across Canada. As well, because the outcome was common (i.e., the majority received magnesium sulphate), we were able to directly estimate the relative risk. However, there are also limitations to our study. First, there were variables with uncertain time points, such as peak BP measurements and proteinuria; however, exclusion of these factors from modelling did not change the significance of the other factors in the final model, and restricting the model to intrapartum and postpartum delivery had lower power. Also, the results are based on a high-risk population of women with pre-eclampsia who were admitted to hospital at <29 weeks; although it is possible that our overall rates of use may be higher than rates of use at term, we do not expect that clinical factors associated with magnesium sulphate would vary with gestational age.

## Conclusions

Most women with pre-eclampsia are being treated with magnesium sulphate for eclampsia prevention. As in Magpie, women are identified, although not in optimal numbers, based on symptoms and severe hypertension, but clinicians are using additional factors on which to base treatment decisions. Future work should focus on which women with pre-eclampsia may benefit most from magnesium sulphate if the drug is not administered to all women with pre-eclampsia for reasons of cost (in all settings) or drug availability (in under-resourced settings).

## Supporting information

S1 File CPN dataset for analysis. (CSV)

S2 File Table A. Definitions of adverse conditions and severe complications of pre-eclampsia in relevant SOGC guidelines.
Table B. Details of serious maternal complications according to severity of pre-eclampsia (SOGC definition) and use of magnesium sulphate or not.
Table C. Sensitivity analyses of determinants included in the final model for magnesium sulphate use in all pre-eclampsia as defined by 2014 SOGC Guidelines.
Table D. Sensitivity analyses using severe antihypertensive therapy to define severe hypertension.
Table E. Sensitivity analyses excluding severe hypertension and heavy proteinuria from the model.
Table F. Sensitivity analyses restricting to intrapartum and postpartum administration of magnesium sulphate.
(DOC)
