# Supplementary material for: Determinants of magnesium sulphate use in women hospitalized at <29 weeks with severe or non-severe pre-eclampsia
Source: PLoS One. 2017 Dec 22;12(12):e0189966. doi: 10.1371/journal.pone.0189966 (PMC5741231; doi:10.1371/journal.pone.0189966)
Supplement: S2 File — Table A. Definitions of adverse conditions and severe complications of pre-eclampsia in relevant SOGC guidelines. Table B. Details of serious maternal complications according to severity of pre-eclampsia (SOGC definition) and use of magnesium sulphate or not. Table C. Sensitivity analyses of determinants included in the final model for magnesium sulphate use in all pre-eclampsia as defined by 2014 SOGC Guidelines. Table D. Sensitivity analyses using severe antihypertensive therapy to define severe hypertension. Table E. Sensitivity analyses excluding severe hypertension and heavy proteinuria from the model. Table F. Sensitivity analyses restricting to intrapartum and postpartum administration of magnesium sulphate. (DOC) [file pone.0189966.s002.doc]

**SUPPLEMENTARY APPENDIX**

| **Table/figure** | **Title** | **Page** |
| --- | --- | --- |
| **Table A** | Definitions of adverse conditions and severe complications of pre-eclampsia | 2 |
| **Table B** | Details of severe maternal complications of pre-eclampsia | 5 |
| **Table C** | Sensitivity analyses of all pre-eclampsia using 2014 SOGC Guidelines | 6 |
| **Table D** | Sensitivity analyses using severe antihypertensive therapy to define severe hypertension | 7 |
| **Table E** | Sensitivity analyses excluding severe hypertension and heavy proteinuria | 8 |
| **Table F** | Sensitivity analyses restricting to intrapartum and postpartum administration of magnesium sulphate | 9 |

**Table A: Definitions of adverse conditions and severe complications of pre-eclampsia in relevant SOGC guidelines.**

|  | **1997 CHS pre-eclampsia** | | **2008 SOGC pre-eclampsia** | | **2014 SOGC pre-eclampsia** | |
| --- | --- | --- | --- | --- | --- | --- |
|  | **Pre-eclampsia (without adverse conditions)** | **Pre-eclampsia (with adverse conditions)** | **Pre-eclampsia** | **Severe pre-eclampsia** | **Pre-eclampsia** | **Severe pre-eclampsia** |
|  |  |  |  |  |  |  |
| **Timing of onset** | - | - | - | <34 wk | - | - |
| **Proteinuria** | ≥0.3g/d in a 24-hour  urine collection | ≥3g/d in a 24-hour urine collection | ≥0.3g/d in a 24-hour  urine collection or ≥30mg/mmol urinary creatinine in a spot (random) urine sample | Heavy proteinuria  (3–5 g/d) | ≥0.3g/d in a 24-hour  urine collection or ≥30mg/mmol urinary creatinine in a spot (random) urine sample | (No criteria for heavy proteinuria) |
|  |  |  |  | One/more adverse condition |  |  |
| **Organ system affected** |  | **One/more adverse condition(s)**  (that increase the risk of severe complications) | **One/more adverse condition(s)**  if there were NO proteinuria |  | **One/more adverse condition(s)**  (that increase the risk of severe complications) |  |
| **CNS** |  | Headache or  visual symptoms | Headache (persistent/new) or visual disturbances |  | Headache or visual symptoms |  |
|  |  | Eclampsia | Eclampsia |  |  | Eclampsia |
|  |  |  | - |  |  | PRES |
|  |  | Cortical blindness or retinal detachment | - |  |  | Cortical blindness or retinal detachment |
|  |  |  | - |  |  | Glasgow coma scale <13 |
|  |  |  | - |  |  | Stroke, TIA, or RIND |
| **Cardiorespiratory** |  | Chest pain/dyspnea | Chest pain/dyspnea |  | Chest pain/dyspnea |  |
|  |  | Severe hypertension | Severe hypertension |  |  | Uncontrolled severe hypertension (over a period of 12hr despite use of three antihypertensive agents) |
|  |  | - | - |  | Oxygen saturation <97% | Oxygen saturation <90%, need for ≥50% oxygen for >1hr, intubation (other than for Caesarean section) |
|  |  | Pulmonary edema | Pulmonary edema |  |  | Pulmonary edema |
|  |  |  | - |  |  | Positive inotropic support |
|  |  |  | - |  |  | Myocardial ischemia or infarction |
| **Hematological** |  | Platelet count <100x10^9^/L | Platelet count <100x10^9^/L |  | Low platelet count | Platelet count <50x10^9^/L |
|  |  |  |  |  | Elevated WBC count |  |
|  |  |  | - |  | Elevated INR or aPTT |  |
|  |  |  | - |  |  | Transfusion of any blood product |
| **Renal** | Elevated serum creatinine |  | Elevated serum creatinine [according to local laboratory criteria] |  | Elevated serum creatinine |  |
|  |  |  | - |  | Elevated serum uric acid |  |
|  |  |  | - |  |  | Acute kidney injury (creatinine >150µm with no prior renal disease); New indication for dialysis |
| **Hepatic** |  | Severe nausea or vomiting | Severe nausea or vomiting |  | Nause or vomiting |  |
|  |  | RUQ or epigastric pain | Persistent abdominal or RUQ pain |  | RUQ or epigastric pain |  |
|  |  | Elevated serum AST, ALT, LDH, or bilirubin | Elevated AST, ALT or LDH [according to local laboratory criteria] *with symptoms* |  | Elevated serum AST, ALT, LDH, or bilirubin |  |
|  |  | Serum albumin <18 g/L | Serum albumin < 20 g/L |  | Low plasma albumin |  |
|  |  |  | - |  |  | Hepatic dysfunction (INR >2 in absence of DIC or warfarin) |
|  |  |  | - |  |  | Hepatic haematoma or rupture |
| **Feto-placental** |  |  | - |  | Non-reassuring FHR |  |
|  |  | IUGR | IUGR |  | IUGR |  |
|  |  | Oligohydramnios | Oligohydramnios |  | Oligohydramnios |  |
|  |  | Absent or reversed end-diastolic flow by Doppler velocimetry | Absent or reversed end-diastolic flow in the umbilical artery by Doppler velocimetry |  | Absent or reversed end-diastolic flow by Doppler velocimetry |  |
|  |  | Suspected placental abruption | Suspected placental abruption |  |  | Abruption with evidence of maternal or fetal compromise |
|  |  |  | Intrauterine fetal death |  |  | Stillbirth |
|  |  |  | - |  |  | Reverse ductus venosus A wave |

*ALT (alanine transaminase), aPTT (activated partial thromboplastin time), AST (aspartate transaminase), CHS (Canadian Hypertension Society), FHR (fetal heart rate), INR (international normalized ratio), IUGR (intrauterine growth restriction), LDH (lactate dehydrogenase), PRES (posterior reversible encephalopathy syndrome), RIND (reversible ischemic neurological deficit), RUQ (right upper quadrant), SOGC (Society of Obstetricians and Gynaecologists of Canada), TIA (transient ischemic attack)*

**Table B: Details of serious maternal complications according to severity of pre-eclampsia (SOGC definition*) and use of magnesium sulphate or not.** Data presented as N (%) or median [IQR].

|  | **Severe pre-eclampsia** | | **Non-severe pre-eclampsia** | |
| --- | --- | --- | --- | --- |
|  | **MgSO4**  **N=131** | **No MgSO4**  **N=43** | **MgSO4**  **N=291** | **No MgSO4**  **N=166** |
| **Serious Maternal complications (one/more)** | 131 (100%) | 43 (100%) | 32 (11.0%) | 16 (9.6%) |
| Third injectable antihypertensive | 8 (6.1%) | 0 | 1 (0.3%) | 0 |
| Myocardial ischemia/infarction | 0 | 0 | 0 | 0 |
| Blindness | 0 | 0 | 0 | 0 |
| Eclampsia | 15 (11.5%) | 2 (4.7%) | 0 | 0 |
| GCS <13 | 0 | 0 | 0 | 0 |
| Stroke | 0 | 1 (2.3%) | 0 | 0 |
| Intubation | 3 (2.3%) | 2 (4.7%) | 1 (0.3%) | 0 |
| Pulmonary Edema | 28 (21.4%) | 6 (14.0%) | 3 (1.0%) | 0 |
| Requirement of >50% O2 | 21 (16.0%) | 3 (7.0%) | 2 (0.7%) | 0 |
| Acute renal failure | 4 (3.1%) | 2 (4.7%) | 1 (0.3%) | 0 |
| Dialysis | 0 | 0 | 0 | 0 |
| Hepatic failure/dysfunction | 9 (6.9%) | 0 | 4 (1.4%) | 0 |
| Hepatic hematoma/rupture | 0 | 0 | 1 (0.3%) | 0 |
| Placental abruption | 12 (9.2%) | 6 (14.0%) | 1 (0.3%) | 3 (1.8%) |
| Maternal blood transfusion | 32 (24.4%) | 6 (14.0%) | 2 (0.7%) | 2 (1.2%) |
| Stillbirth | 46 (35.1%) | 24 (55.8%) | 2 (0.7%) | 4 (2.4%) |

*GCS (Glasgow Coma Score), MgSO4 (magnesium sulphate), SOGC (Society of Obstetricians and Gynaecologists of Canada)*

**as defined in Table S2*

**Table C**: Sensitivity analyses of determinants included in the final model for magnesium sulphate use in all pre-eclampsia as defined by 2014 SOGC Guidelines*

| **Determinants** | **Adjusted RR [95% CI]** | **PAR% [95% CI]** |
| --- | --- | --- |
| **Demographic and clinical characteristics in index pregnancy** |  |  |
| Maternal Age (yr) |  |  |
| ≤24 | 1.11 [1.05, 1.17] | 0.07 [0.00, 3.01] |
| 25-29 | 1.03 [0.96, 1.11] | 2.30 [-2.37, 3.99] |
| 30-34 | Reference | Reference |
| ≥35 | 1.01 [0.92, 1.11] | 0.98 [-4.34, 4.43] |
| Pre-existing hypertension | 0.93 [0.86, 1.00] | -1.83 [-4.64, 0.69] |
| Nulliparity | 1.08 [1.00, 1.16] | 4.91 [-2.69, 11.87] |
| Singleton pregnancy | 1.23 [1.00, 1.53] | 17.28 [-5.01, 30.82] |
| **Type of pre-eclampsia** |  |  |
| Severe | 1.00 [0.94, 1.07] | 0.27 [-2.87, 2.50] |
| Non-severe | Reference | Reference |
| **Pre-eclampsia severity criteria** |  |  |
| Severe hypertension (sBP ≥160 or dBP ≥110) | 1.34 [1.08, 1.67] | 22.04 [7.11, 34.35] |
| Heavy proteinuria (≥3+ or ≥3.0g/d) | 1.12 [1.01, 1.24] | 7.88 [0.56, 16.21] |
| Delivery for maternal symptoms or sign(s) of pre-eclampsia | 2.73 [1.30, 5.72] | 48.73 [40.57, 56.52] |
| Fetal syndrome of pre-eclampsia† | 0.94 [0.89, 0.99] | -4.49 [-10.05, 2.35] |
| **Maternal interventions prescribed** |  |  |
| Maternal transport prior to delivery | 1.22 [1.12, 1.32] | 9.90 [4.77, 14.56] |
| Interventionist care | 1.21 [1.17, 1.25] | 3.46 [1.90, 4.99] |
| Antenatal corticosteroids | 1.31 [1.09, 1.58] | 19.99 [9.09, 32.48] |
| Spontaneous labor initiation | 0.72 [0.26, 2.01] | -1.97 [-10.97, 0.67] |

*dBP (diastolic blood pressure), PAR% (population attributable risk percent), RR (relative risk), sBP (systolic blood pressure), SOGC (Society of Obstetricians and Gynaecologists of Canada)*

** Variables highlighted in yellow demonstrated significant, independent associations with magnesium sulphate use.*

†*Includes one or more of abnormal Doppler of umbilical artery, oligohydramnios, intrauterine growth restriction, birthweight <10^th^ centile, and stillbirth*

**Table D: Sensitivity analyses using severe antihypertensive therapy to define severe hypertension***

| **Determinants** | **Adjusted RR [95% CI]** | **PAR% [95% CI]** |
| --- | --- | --- |
| **Demographic and clinical characteristics in index pregnancy** |  |  |
| Maternal age (yr) |  |  |
| ≤24 | 1.11 [1.06, 1.17] | 1.48 [-0.30, 2.84] |
| 25-29 | 1.03 [0.97, 1.09] | 0.61 [-2.50, 3.32] |
| 30-34 | Reference | Reference |
| ≥35 | 1.02 [0.93, 1.11] | 0.51 [-4.23, 4.17] |
| Pre-existing hypertension | 0.92 [0.86, 1.00] | -1.90 [-5.26, 1.08] |
| Nulliparity | 1.09 [1.01, 1.17] | 5.27 [-0.77, 11.74] |
| Singleton pregnancy | 1.28 [1.05, 1.56] | 20.16 [5.12, 36.98] |
| **Pre-eclampsia severity criteria** |  |  |
| Severe antihypertensive therapy ∫ | 1.21 [1.15, 1.28] | 5.97 [3.32, 8.48] |
| Heavy proteinuria (≥3+ or ≥3.0g/d) | 1.13 [1.02, 1.25] | 8.55 [-0.56, 15.99] |
| Delivery for adverse condition of maternal symptoms of pre-eclampsia or maternal sign(s) of pre-eclampsia | 2.74 [1.34, 5.63] | 48.84 [41.30, 57.37] |
| Severe maternal complications | 1.03 [0.96, 1.10] | 0.96 [-1.96, 3.47] |
| Fetal syndrome of pre-eclampsia† | 0.93 [0.88, 0.98] | -4.93 [-11.93, 0.88] |
| **Maternal interventions prescribed** |  |  |
| Maternal transport prior to delivery | 1.22 [1.12, 1.32] | 9.82 [4.87, 14.76] |
| Interventionist care | 1.18 [1.15, 1.22] | 3.00 [1.50, 4.63] |
| Antenatal corticosteroids | 1.30 [1.09, 1.55] | 19.49 [7.48, 31.85] |
| Spontaneous labor initiation | 0.69 [0.28, 1.75] | -2.23 [-10.26, 0.26] |

*dBP (diastolic blood pressure), PAR% (population attributable risk percent), RR (relative risk), sBP (systolic blood pressure), SOGC (Society of Obstetricians and Gynaecologists of Canada)*

** Variables highlighted in yellow demonstrated significant, independent associations with magnesium sulphate use.*

*∫ Includes use of parenteral hydralazine or labetalol, or nifedipine capsules or intermediate-acting tablets.*

†*Includes one or more of abnormal Doppler of umbilical artery, oligohydramnios, intrauterine growth restriction, birthweight <10^th^ centile, and stillbirth*

**Table E: Sensitivity analyses excluding severe hypertension and proteinuria from the model.**

| **Determinants** | **Adjusted RR [95% CI]** | **PAR% [95% CI]** |
| --- | --- | --- |
| **Demographic and clinical characteristics in index pregnancy** |  |  |
| Maternal age (yr) |  |  |
| ≤24 | 1.13 [1.06, 1.20] | 1.64 [-0.11, 3.11] |
| 25-29 | 1.04 [0.97, 1.11] | 0.86 [-2.30, 3.39] |
| 30-34 | Reference | Reference |
| ≥35 | 1.01 [0.92, 1.11] | 0.32 [-3.60, 3.95] |
| Pre-existing hypertension | 0.94 [0.87, 1.01] | -1.51 [-4.57, 0.85] |
| Nulliparity | 1.07 [1.00, 1.15] | 4.49 [-2.21, 10.17] |
| Singleton pregnancy | 1.30 [1.04, 1.62] | 20.95 [4.45, 38.12] |
| **Pre-eclampsia severity criteria** |  |  |
| Delivery for adverse condition of maternal symptoms of pre-eclampsia or maternal sign(s) of pre-eclampsia | 2.90 [1.31, 6.39] | 50.32 [42.56, 57.94] |
| Severe maternal complication | 1.04 [0.97, 1.11] | 1.33 [-2.11, 4.28] |
| Fetal syndrome of pre-eclampsia† | 0.93 [0.88, 0.98] | -4.94 [-12.40, 1.24] |
| **Maternal interventions prescribed** |  |  |
| Maternal transport prior to delivery | 1.22 [1.12, 1.33] | 10.06 [5.73, 14.77] |
| Interventionist care | 1.20 [1.16, 1.24] | 3.31 [1.83, 4.84] |
| Antenatal corticosteroids | 1.29 [1.06, 1.57] | 19.13 [6.49, 30.44] |
| Spontaneous labor initiation | 0.67 [0.26, 1.73] | -2.49 [-11.25, 0.13] |

*dBP (diastolic blood pressure), PAR% (population attributable risk percent), RR (relative risk), sBP (systolic blood pressure), SOGC (Society of Obstetricians and Gynaecologists of Canada)*

** Variables highlighted in yellow demonstrated significant, independent associations with magnesium sulphate use.*

†*Includes one or more of abnormal Doppler of umbilical artery, oligohydramnios, intrauterine growth restriction, birthweight <10^th^ centile, and stillbirth*

**Table F: Sensitivity analyses restricting to intrapartum and postpartum administration of magnesium sulphate.**

| **Determinants** | **Adjusted RR [95% CI]** | **PAR% [95% CI]** |
| --- | --- | --- |
| **Demographic and clinical characteristics in index pregnancy** |  |  |
| Maternal age (yr) |  |  |
| ≤24 | 1.22 [0.80, 1.88] | 2.67 [-1.45, 5.72] |
| 25-29 | 1.00 [0.65, 1.53] | -0.03 [-6.55, 4/74] |
| 30-34 | Reference | Reference |
| ≥35 | 1.10 [0.77, 1.57] | 2.96 [-4.77, 9.94] |
| Pre-existing hypertension | 0.90 [0.58, 1.42] | -2.41 [-9.17, 2.10] |
| Nulliparity | 1.04 [0.72, 1.50] | 2.53 [-11.38, 14.58] |
| Singleton pregnancy | 1.51 [0.34, 6.65] | 31.08 [-0.45, 57.51] |
| **Pre-eclampsia severity criteria** |  |  |
| Severe hypertension (sBP ≥160 or dBP ≥110) | 1.11 [0.61, 2.01] | 8.54 [-15.19, 29.57] |
| Heavy proteinuria (≥3+ or ≥3.0g/d) | 1.01 [0.67, 1.53] | 0.88 [-15.41, 16.92] |
| Delivery for maternal symptoms or sign(s) of pre-eclampsia | 3.18 [0.30, 34.09] | 52.69 [38.54, 63.16] |
| Severe maternal complications | 0.92 [0.72, 1.19] | -2.96 [-11.05, 3.51] |
| Fetal syndrome of pre-eclampsia† | 0.83 [0.60, 1.49] | -13.36 [-29.72, -0.24] |
| **Maternal interventions prescribed** |  |  |
| Maternal transport prior to delivery | 1.04 [0.72, 1.49] | 1.92 [-6.91, 10.92] |
| Interventionist care | 1.14 [0.87, 1.48] | 2.38 [-1.27, 5.93] |
| Antenatal corticosteroids | 1.82 [0.64, 5.18] | 37.98 [18.35, 52.37] |
| Spontaneous labour initiation | 1.07 [0.37, 3.12] | 0.35 [-3.39, 2.34] |

*BP (blood pressure), dBP (diastolic BP), PAR% (population attributable risk), RR (relative risk), sBP (systolic BP)*

** Variables highlighted in yellow demonstrated significant, independent associations with magnesium sulphate use.*

*† Includes one/more of abnormal Doppler of umbilical artery, oligohydramnios, intrauterine fetal growth restriction, birthweight <10^th^ centile, and stillbirth*
